# Chronic gallbladder wall thickening: Is it always malignancy?

**DOI:** 10.4102/sajr.v24i1.1844

**Published:** 2020-05-18

**Authors:** Anjuna Reghunath, Suchana Kushvaha, Rohini G. Ghasi, Geetika Khanna, Apurva Surana

**Affiliations:** 1 Department of Radiodiagnosis, Vardhman Mahavir Medical College and Safdarjung Hospital, New Delhi, India

**Keywords:** xanthogranulomatous cholecystitis, gallbladder cancer, ultrasound, computed tomography, magnetic resonance imaging

## Abstract

Gallbladder wall thickening, associated with features like perforation, fistula formation and invasion of adjacent organs, is often assumed to be malignant. Xanthogranulomatous cholecystitis (XGC) causes gallbladder wall thickening with similar aggressive features and closely mimics gallbladder carcinoma clinically, radiologically and surgically. Differentiating between these two is crucial for management as misdiagnosis of gallbladder cancer can lead to unnecessary radical surgery. We report a case of chronic gallbladder wall thickening, initially suspected to be malignant, but subsequently diagnosed as XGC.

## Introduction

Xanthogranulomatous cholecystitis (XGC) is a lesser known variant of chronic cholecystitis which causes gallbladder (GB) wall thickening with aggressive features similar to GB carcinoma, such as GB wall perforation, fistula formation and invasion of adjacent organs. Xanthogranulomatous cholecystitis is a chronic, focal or diffuse, destructive, fibro-inflammatory disease of the GB that results from intramural accumulation of foamy macrophages and inflammatory cells, with proliferative fibrosis in later stages.^1^ The pathogenesis of this uncommon condition is not fully understood.^2^ Xanthogranulomatous cholecystitis is often confused with GB carcinoma because of its aggressive nature and overlapping clinical, imaging and surgical features.^3^ It is crucial to differentiate between these two entities from a management perspective since a misdiagnosis of XGC as GB cancer can lead to unnecessary radical surgery.

The purpose of this article was to highlight the fact that not every chronic GB wall thickening is malignant and to review the distinguishing imaging features of XGC.

## Case report

A 74-year-old woman presented with right upper quadrant pain for 6 months. There was neither a history of fever or jaundice nor any significant past history. Clinical examination was insignificant, with a negative Murphy’s sign. Her liver function tests were within normal limits. An ultrasound abdomen conducted elsewhere had revealed thickening of GB wall, and the patient was therefore referred for a contrast-enhanced computed tomography (CECT) to exclude any underlying malignancy.

**FIGURE 1:**
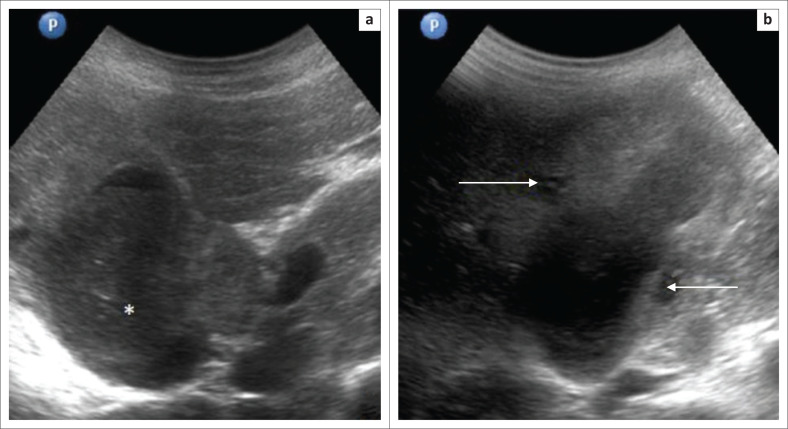
(a) Ultrasound abdomen revealing a distended gallbladder filled with echogenic sludge (asterisk), (b) with asymmetrical wall thickening and intramural hypoechoic nodules (arrows).

A pre-computed tomography (CT) ultrasound scan, performed at our institute, demonstrated a distended GB, filled with echogenic sludge (Figure 1a). Diffuse, asymmetrical, hyperechoic wall thickening was noted with two well-defined hypoechoic intramural nodules within the wall of GB (Figure 1). No comet-tail artefacts were seen in the wall.

**FIGURE 2:**
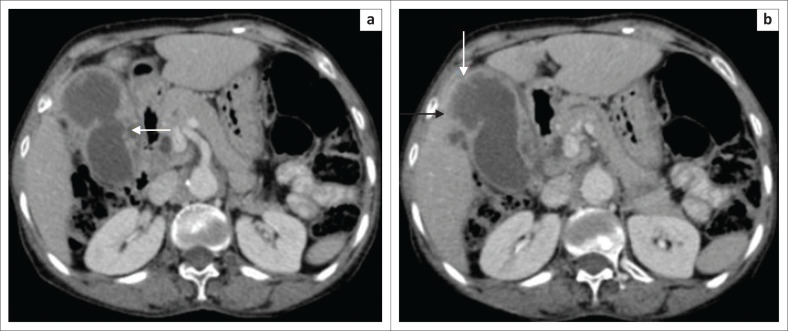
(a) Nodules appear hypodense on contrast-enhanced computed tomography images (arrow). (b) Focal breach in mucosal lining (white arrow) and indistinctiveness with adjacent liver noted (black arrow).

Contrast-enhanced computed tomography revealed homogeneous enhancement of the thickened GB wall with multiple intramural cystic areas (Figure 2a). Mucosal enhancement was relatively well preserved, except for a focal breach in the region of the body of the GB adjacent to the liver (Figure 2b); however, fat planes between the remaining organs were maintained. Minimal fat stranding was noted surrounding the GB fundus. No gallstones, significant periportal lymphadenopathy or intrahepatic biliary radical dilatation was observed. Based on the ultrasound and CECT findings, the differential diagnoses considered were XGC and GB carcinoma.

**FIGURE 3:**
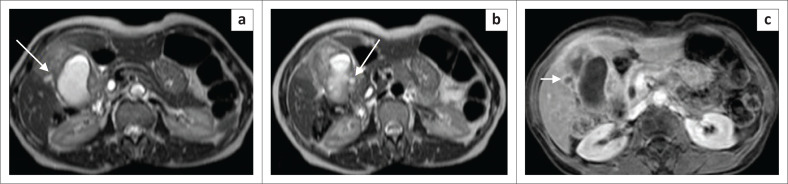
(a and b) Axial T2W magnetic resonance imaging images demonstrating hyperintense intramural nodules (arrows), which on post-contrast T1W sequence (c) shows peripheral enhancement (black arrow), suggestive of a microabscess.

We further performed magnetic resonance imaging (MRI) of the abdomen to differentiate between XGC and GB cancer. Magnetic resonance imaging demonstrated a few intramural T2 hyperintense nodules within the wall of GB, confirming the diagnosis of XGC (Figure 3a–c). After intravenous gadolinium administration, the GB wall showed uniform enhancement with focal discontinuous mucosal enhancement. Although the adjacent liver had shown T2 hyperintense signal suggestive of oedema, post-contrast images indicated a maintained boundary between the enhanced GB wall and liver (Figure 3), suggesting inflammatory changes rather than invasion.

**FIGURE 4:**
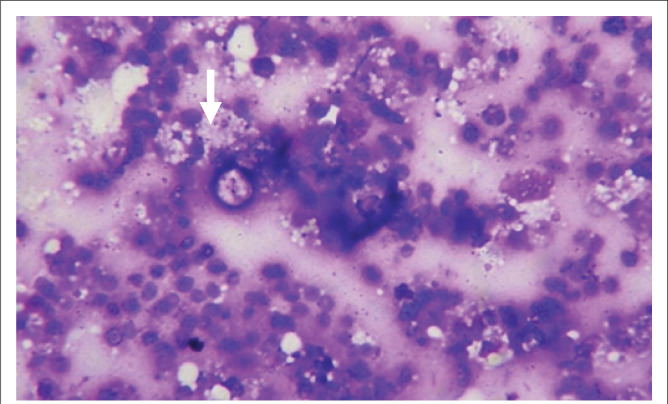
Fine needle aspiration cytology specimen from the fundus showing foamy macrophages (arrow) and polymorphs on May–Grunwald–Giemsa stain, consistent with the diagnosis of xanthogranulomatous cholecystitis.

Tissue sampling was obtained with ultrasound-guided fine needle aspiration cytology (FNAC) from the fundus of GB. Histology showed polymorphs and foamy histiocytes, in keeping with XGC (Figure 4). The patient subsequently underwent an open simple cholecystectomy.

### Ethical consideration

Consent was taken from the patient to include the data in the study.

## Discussion

Xanthogranulomatous cholecystitis is an uncommon variant of chronic cholecystitis, characterised by intramural xanthogranulomatous (accumulation of lipid-laden macrophages) inflammation (with both acute and chronic inflammatory cells) of the GB.^1^ Its incidence varies from 0.7% to 10%^1^ and men are affected twice as commonly as women, with a peak incidence occurring around the sixth to seventh decade of life.^1^ This case describes XGC in a female patient, an unusual presentation.

The most important association with XGC is cholelithiasis, seen in approximately 66.6% of patients.^4^ The hypothesis behind the pathogenesis of this condition is GB outflow or cystic duct obstruction by calculi, leading to a rupture of the Rokitansky–Aschoff sinuses, causing bile leakage into the wall of the GB.^5^ Bile is then engulfed by macrophages and foamy histiocytes, resulting in a chronic granulomatous response, microabscess formation, subsequent wall fibrosis and scarring. This results in a high complication rate (up to 32%)^5^ and includes GB perforation, adhesions, peritoneal scar formation and fistulous tracts to the stomach, duodenum, hepatic flexure or transverse colon and anterior abdominal wall.^5^

Xanthogranulomatous cholecystitis presents clinically as chronic (88%) or acute cholecystitis (22%).^1^ Common clinical features include abdominal pain, obstructive jaundice or cholangitis.^1^ Occasionally, there may be a palpable mass or a positive Murphy’s sign. Laboratory parameters are usually normal, with no associated specific liver function test discordance.^6^ Xanthogranulomatous cholecystitis can have co-existing GB carcinoma in 8.5% – 30.5% of cases or infection with *Escherichia coli, Klebsiella, Enterococcus, Pseudomonas* or *Staphylococcus.*^1^

Imaging modalities play a major role in the detection of XGC. The typical sonographic findings include diffuse, symmetrical, hyperechoic wall thickening, intramural hypoechoic nodules, associated cholelithiasis or choledocholithiasis.^5^ On CECT, the wall thickening is always > 3mm and shows homogeneous enhancement^3^. Intramural hypoattenuating nodules are present in 85% of cases.^6^ These nodules may represent xanthogranulomas or microabscesses depending on the phase of inflammation. Luminal surface enhancement (LSE) with continuous mucosal lines is seen in 66% of cases, and a focal breach in the mucosal line is not uncommon.^1^ Pericholecystic fat stranding, blurring of the interface with liver, oedema, transient hepatic attenuation differences or early enhancement may be appreciated in the adjacent liver parenchyma.^4^ Zhao et al.^6^ observed that the co-existence of at least four out of the five CT features (diffuse GB wall thickening, hypoattenuating intramural nodules, continuous mucosal line, LSE and gallstones) was found in 80% cases of histologically proven XGC.^6^

On dynamic contrast-enhanced MRI, areas of T2 isointensity showing early and strong delayed enhancement suggest xanthogranulomas, whereas high T2 signal lesions without enhancement suggest microabscesses.^1^ On diffusion weighted imaging, XGCs can show hyperintensity with corresponding hypointensity on Apparent Diffusion Coefficient (ADC) map; however, restriction is more common in carcinoma (mean ADC:1.076) than in XGC (mean ADC: 1.637).^7^ In-phase/out-phase chemical shift imaging demonstrates fat in the GB wall in about 77.7% of cases,^8^ suggesting the presence of fat-containing xanthogranulomas in the wall of the GB.

Common differential diagnoses of XGC include carcinoma of the GB and adenomyomatosis.^6,9^ Features differentiating XGC from GB cancer are presented in Table 1. Both XGC and adenomyomatosis demonstrate wall thickening with sonographic hypoechoic intramural nodules and gallstones.^5^ Rokitansky–Aschoff sinuses are also visualised on T2-weighted MRI as the ‘Pearl necklace sign’ and cholesterol crystals within them show comet-tail artefact.^5^ Complications due to scar formation in XGC are typically absent in adenomyomatosis.^5^ Another differential diagnosis of XGC includes actinomycosis of the GB, which presents as an infiltrative mass with multiple abscesses, draining sinuses and dense fibrous tissue, making differentiation challenging, except when associated with abdomino-pelvic actinomycosis.^10^

**TABLE 1:** Differences in imaging features of carcinoma gallbladder and xanthogranulomatous cholecystitis.

Carcinoma gallbladder	Xanthogranulomatous cholecystitis
Focal, asymmetric wall thickening	Diffuse, symmetric wall thickening
Interrupted mucosal lining	Continuous mucosal lining more common than interrupted
No intramural nodules	Intramural hypoattenuating nodules
Direct macroscopic infiltration of mass into adjacent liver	Absence of macroscopic hepatic invasion
Intrahepatic biliary radical dilatation may be associated	Intrahepatic biliary radical dilatation usually absent
Significant heterogeneous or necrotic periportal or retroperitoneal lymph nodes or distant metastasis	Absence of significant lymphadenopathy and metastasis

Other conditions with overlapping features, such as wall thickening and mass-forming lesions of the GB with adhesions, are collectively labelled as inflammatory pseudo tumours of the GB. These include inflammatory myofibroblastic tumours, inflammatory tumours developing as a foreign body reaction and Immunoglobulin G4 (IgG4)-related cholecystitis.^11^ In a study performed by Hong et al.^12^ in 2018, significant concurrence of histopathologic features of IgG4-related disease like dense lymphoplasmocytic and IgG4 plasma cell infiltration, storiform fibrosis and obliterative phlebitis was observed in surgically resected specimens of GB, with pathologic evidence suggesting XGC. The authors concluded that the conditions may co-exist, especially when IgG4-related disease involves other organs.^12^ A comparison of the imaging features in various inflammatory causes of gallbladder wall thickening is presented in Table 2.

**TABLE 2:** Comparison of imaging features in inflammatory causes of gallbladder wall thickening.

Condition	Intramural nodules	Gallstones	Adhesion/fistulisation	Biliary radical dilatation	Enhancement pattern	Systemic involvement
Xanthogranulomatous cholecystitis	Seen	Common	Common	-	Type 1	-
Adenomyomatosis	Intramural nodules with comet-tail artefacts	Common	Uncommon	-	Type 3	-
Actinomycosis	-	-	Common	-	-	Pelvic disease
IgG4-related disease	-	-	Uncommon	Common	-	Multi-organ involvement
Calculous cholecystitis	-	Present	Uncommon	May be seen if associated choledocholithiasis present	Type 4 in chronic and type 5 in acute calculous cholecystitis	-

Contrast-enhanced computed tomography is the best modality for the evaluation of GB wall thickening and a systematic approach may be beneficial in arriving at an accurate diagnosis, as follows:

1. Is the thickening focal or diffuse? Focal thickening may be seen in GB carcinoma, focal XGC or focal adenomyomatosis. Pathologies with diffuse thickening may or may not be associated with cholelithiasis. Gallstones are associated with acute or chronic cholecystitis, GB cancer, XGC and adenomyomatosis, while calculi are usually absent in actinomycosis, IgG4-related disease, acalculous cholecystitis and GB wall thickening secondary to hepatitis, liver cirrhosis, congestive heart failure, renal failure, pancreatitis or Dengue fever.
2. Is the diffuse thickening symmetrical or asymmetrical? Asymmetrical thickening is more common in malignancies as compared to inflammatory conditions.
3. The presence of intramural nodules: intramural nodules may be seen in XGC, as well as in adenomyomatosis. However, ultrasound would distinguish between the two by the presence of comet-tail artefacts.
4. Mucosal enhancement pattern: Kim et al.^13^ described five patterns of GB wall enhancement in 2008. Type 1 is a homogeneously or heterogeneously enhancing thick single layer pattern, seen in XGC and GB carcinoma. Type 2 pattern shows intense inner layer enhancement with a weakly enhanced or unenhanced outer layer, seen in GB cancer. In type 3 pattern, the inner layer enhances similar to hepatic parenchyma with a weakly enhancing outer layer, as seen in adenomyomatosis. The type 4 pattern is typically seen in chronic calculous cholecystitis, with a weakly enhancing, fuzzy, inner layer and a thin non-enhancing outer layer along with a collapsed lumen. However, this pattern may also be seen in cirrhosis and viral hepatitis.^14^ The type 5 pattern is depicted by a weakly enhancing, fuzzy, inner layer with a non-enhancing thick outer layer of submucosal oedema, as seen in acute calculous cholecystitis and Dengue fever.^13,14^
5. Secondary bile duct dilatation: seen in GB cancer and IgG4-related disease.
6. Pericholecystic inflammation, adhesions, fistulisation with bowel: GB cancer, XGC, actinomycosis.
7. Liver invasion and nodal involvement: common in GB carcinoma.
8. Involvement of other organs: metastasis in GB cancer, pelvic disease in actinomycosis and multi-organ involvement in IgG4 disease.

Ideal management of XGC is open cholecystectomy with excision of the inflammatory tissue. This is not the case for GB carcinoma, which requires a more radical wider excision for a tumour-free resection margin and regional lymph node dissection.^7^

## Conclusion

Xanthogranulomatous cholecystitis closely simulates GB carcinoma clinically and radiologically. A less aggressive surgical approach like simple cholecystectomy is warranted in XGC and its correct diagnosis may avert aggressive surgeries like Whipple’s or partial or segmental hepatectomy.^2^ However, one should remember that XGC may co-exist with GB cancer as well and a definitive diagnosis necessitates histopathologic analysis. A combination of clinico-radiological factors combined with intra-operative frozen-section examination^9^ aids in deciding further management and planning the surgical approach, thus improving patient care.
